# Addressing the urban congestion challenge based on traffic bottlenecks

**DOI:** 10.1098/rsta.2024.0095

**Published:** 2024-11-13

**Authors:** Efrat Blumenfeld Lieberthal, Nimrod Serok, Jinxiao Duan, Guanwen Zeng, Shlomo Havlin

**Affiliations:** ^1^ School of Architecture, Tel Aviv University, Tel Aviv 6997801, Israel; ^2^ School of Traffic and Transportation, Beijing Jiaotong University, Beijing 100044, People’s Republic of China; ^3^ School of Systems Science, Beijing Jiaotong University, Beijing 100044, People’s Republic of China; ^4^ Physics Department, Bar Ilan University, Ramat Gan 5290002, Israel

**Keywords:** smart transportation, urban planning, traffic bottlenecks

## Abstract

Historically, urban congestion and street life quality depended on city network hierarchies, shaped by land use and street layout. Yet navigation apps have shifted focus to travel time as the key route selection factor, challenging traditional urban structures. We review the development of an innovative approach to urban traffic management that leverages real-time data for the identification and analysis of traffic bottlenecks. This approach, combined with urban planning, aims to improve traffic flow and tackle modern urban challenges. It includes real-time bottleneck detection and cost, congestion analysis and designing a decentralized traffic management system that can serve planners. Based on complex system principles, it promises dynamic traffic optimization, merging urban planning with digital advancements. The research demonstrates the potential of different applications of the proposed methodologies to predict significant congestion from early bottleneck formation, offering urban planners a powerful toolset for reasserting their role in shaping the urban experience. This article posits that a nuanced understanding of traffic dynamics, coupled with advanced traffic management technologies, can restore the influence of urban planning in the digital era, fostering more liveable, equitable and efficient urban environments.

This article is part of the theme issue ‘Co-creating the future: participatory cities and digital governance’.

## 1. Introduction

Since 2008, over half of the world’s population lives in cities, a number projected to reach 68% by 2050. Urbanization boosts economic growth, as cities, being centres of productivity and innovation, thrive on economies of scale and concentrated human capital. Urban theories have evolved to prioritize interactions, networks and processes over mere location, seeing cities as networks of both tangible and intangible exchanges [1]. Urban planning integrates diverse relationships to create spatial reality, requiring a synthesis of theory and practice and recognizing individual complexity as central to urban complexity.

Urban planning, land use and transportation systems are intrinsically linked, influencing urban movement, development and organization [2]. These connections affect location exposure, business expansion and the demand for transportation [3]. Urban transportation not only responds to but also shapes land use, impacting office and residential locations, land prices and urban density. Integrating land-use planning with transportation systems is crucial for sustainable urban mobility, a consensus among researchers, professionals, governments and the private sector [2]. Traditionally, planners used static transportation planning to shape city areas. High streets were designed as wide roads for commerce, while residential areas had narrow streets and cul-de-sacs for local traffic only. This approach guided drivers’ navigation using the primary road network as their route choice strategy [4,5].

With rapid urbanization and vehicle usage increase, urban areas face escalating traffic congestion [6,7]. Congestion costs extend beyond traffic delays, encompassing increased travel times, fuel consumption, pollution, socio-economic disparities, parking shortages and health concerns. It also affects urban transport equity, with unequal access, higher accident risks and greater exposure to pollution and noise, disproportionately impacting pedestrians and cyclists [8,9]. Transport equity research, dealing with the unequal distribution of benefits and burdens, highlights the need for urban transport justice, advocating for a just distribution of transport benefits and burdens [9]. The impact on residents, from health issues to reduced safety, underscores the importance of reevaluating urban planning and transport policies to prioritize equitable, healthy urban environments. Thus, there is a substantial body of research in various disciplines that aims at reducing traffic congestion, in general, and in urban areas, in particular.

The present review describes work that seeks to expand the scope of urban planning practices and to align it with contemporary perspectives and the accompanying technological advancements. Recently, there has been a growing momentum in leveraging real-time urban traffic data to mitigate traffic congestions [10–12]. Thus, we believe planners should be empowered with modern tools to manage the complexities of urban planning effectively, as existing apps like Waze and Google Maps use real-time data (location, speed) to offer users the best routes, adjusting for traffic, roadblocks and other issues. However, such technologies also redirect traffic to quieter streets, disrupting traditional planning and emphasizing time efficiency over urban character. This shift challenges traditional urban planning by prioritizing time efficiency over urban design. Consequently, the relevance of top-down planning diminishes as the immediate needs for mobility and efficiency supersede intended urban ambiences and local traffic considerations.

This research emphasizes the need for a balance between algorithmic optimization and planners’ expertise to ensure urban planning addresses both traffic dynamics and urban life quality, not just minimizing travel time. It aims to make urban planning more relevant by utilizing real-time data for urban analysis and interventions. The key question is as follows: How can real-time traffic data improve urban planning, offering insights for better traffic management and tools that enhance comprehensive planning’s impact on urban traffic organization?

More specifically, this work aims to enhance urban planning by analysing traffic bottlenecks and their effects on cities, proposing tools for integrating a broader understanding of urban dynamics. By emphasizing the dynamic interplay between bottom-up and top-down decisions informed by real-time data, we suggest that urban planning should be flexible and responsive, fostering a feedback loop that adapts to and influences the evolving urban environment.

## 2. State of the art

The evolution of complex systems science reflects a shift from centralized, top-down models to decentralized, bottom-up constructions. This field examines interconnected elements within systems, focusing on feedback loops and phase transitions that dictate system behaviour and state changes studying problems across diverse fields.

Cities are complex systems that can be regarded as interdependent and interconnected networks representing transportation, economy, society, politics and culture, forming a web of connections that define urban mobility dynamics [6,13,14]. They evolve through self-organization and interactions across multiple scales, driven by both bottom-up and top-down forces [15,16], and their dynamic nature, shaped by various agents including people, economic forces and political aspirations, creates intricate networks that define urban life. Recognizing cities as complex systems highlights the importance of adaptability and flexibility in urban planning, balancing top-down guidance with the potential for bottom-up influence facilitated by technological advancements and connectivity [17].

From its early evolution to the present, urban planning integrates traffic management with land use and spatial organization, reflecting and shaping movement within cities. Modern planning seeks to include all aspects of urban life and efficiency, with recent emphasis on integrating land use and transportation to foster efficient, sustainable urban mobility and preserve diverse urban experiences [2,18].

The challenges of understanding the complex congestion propagation have stimulated extensive traffic flow approaches to modelling and understanding urban traffic dynamics [19,20]. Attention was also paid to understanding the jam formation for the known causes, including the queue model [21], the lane-changing model [22,23] and the cell transmission model [24,25]. Recently, emerging theories from other natural systems have been borrowed and applied to traffic systems to analyse the propagation of traffic congestions across networks, exemplified by the cascading failure models [26,27], epidemic models [28,29] and congestion tree method [30,31]. Studies of traffic condition prediction [32–35] and travel demand control [36–40] also considered congestion propagation and suggested that understanding such spatiotemporal dynamics, especially their bottlenecks, could be effective for forecasting them and thus have the potential to prevent congestions from spreading [41].

### (a) Urban traffic management systems

The first traffic signal system, operating on a fixed-timer basis, was established in Houston in 1922, marking the onset of the second generation of urban traffic control systems. In the 1950s, coordination between intersections led to the concept of the ‘Green Wave’, synchronizing green lights along main arteries to enable continuous travel [42]. Advancements in computer technology allowed for more sophisticated systems, capable of adjusting green light timing based on sensor data detecting waiting vehicles [43]. Intersections were divided into smaller zones for local timing optimization, adjusting cycle time, split time and time offset to enhance traffic flow [44]. Cycle time refers to the duration of one complete set of driving phases at an intersection, split time allocates green light time for each phase and time offset delays green lights between adjacent intersections to ensure smooth traffic flow [44].

Fixed-time plans allow for creating green waves, giving predefined priorities and responding to pre-scheduled events, but they cannot react to unforeseen events like accidents [42]. These plans can quickly become outdated, especially in rapidly growing traffic areas, losing effectiveness by roughly 3% annually without regular updates [45]. The development of more adaptable strategies to match road supply with real-time demand can be categorized into centralized synchronized [44,46] and decentralized systems, reflecting technological advancements and a deeper understanding of urban congestion dynamics [44].

Self-organized traffic control offers a scalable, decentralized solution, enhancing coordination between intersections while reducing computational costs and simplifying implementation. It features two coordination types: *active coordination* relies on interdependent decision-making, using multi-agent reinforcement learning to adjust traffic signals based on collective conditions and strategies [47,48]. *Circumstantial coordination* allows intersections to make independent decisions influenced indirectly by nearby intersections through shared sensor data, promoting a form of coordination without direct interaction.

Centralized and current decentralized traffic control methods face limitations: centralized approaches can ease congestion in small areas, while decentralized ones may cause conflicts at intersections, making large-scale optimal control a complex, NP-hard problem. This complexity arises from the need to manage a self-organizing system where interactions between numerous elements lead to emergent order, challenging real-time optimal solution finding [49]. To address these challenges, a dynamic optimization process that adapts to changes through local interactions is required. A comprehensive strategy, leveraging advanced detection and communication technologies, is necessary for the next evolution in traffic management, requiring a strategic view of the urban network as a whole [44,50]. This review focuses on recent work that utilizes complex network theory to examine the intricate properties of such systems.

Network research spans multiple disciplines, highlighting the versatility of complex networks in understanding systems across sociology, biology, technology and more, using a framework where nodes represent elements and edges denote interactions [14,51,52]. This approach has significantly contributed to various fields, emphasizing the interconnectedness and interdependence of complex systems through network representations. Additionally, the concept of multi-layer networks has emerged, focusing on the interdependencies between different networks, crucial in analysing infrastructure systems and understanding cascade failures [53–55]. This broader perspective underlines the importance of considering complex systems as interconnected and interdependent networks for comprehensive analysis and for problem-solving across diverse fields.

Research has examined various public transportation systems—airlines, marine lines, trains, buses and underground trains—highlighting the importance of network topology in urban planning [56–59]. Studies have also explored the dynamic nature of urban movement, including bike-sharing systems and commuting patterns, underscoring the influence of street network topology on traffic volumes and urban dynamics [60–66]. However, the dynamic aspects of traffic flow and its systemic urban effects are often overlooked, indicating a need for further exploration.

Traffic bottleneck identification has evolved from freeway analysis to urban areas to now using big data for near real-time detection. Freeway studies [67] differ from urban bottleneck identification [30,68] due to urban areas’ complex patterns. Big data have enabled methodologies like dynamic Bayesian networks and complex network theory for identifying and analysing urban bottlenecks [30,67], including causal relationship modelling between traffic sensors [67]. Despite advancements, a gap exists in real-time, holistic traffic management that considers individual vehicle impacts [44]. Innovative approaches like percolation processes have been applied to identify critical urban traffic bottlenecks [69], offering insights into traffic dynamics and street morphology correlations. Thus, here we review recent research that aims to develop new planning tools by understanding the relationship between urban street networks and traffic dynamics, highlighting the potential for smart congestion pricing and traffic volume management.

## 3. Identification and prioritization of urban traffic congestion

This article provides a comprehensive view of four studies [31,70–72] that explore the feasibility of detecting and prioritizing urban traffic bottlenecks through an innovative identification methodology. Initially, the foundational principles of this methodology are delineated, underscoring its utility in the analysis of substantial real-world urban traffic data. Subsequently, this article illustrates the methodology’s capacity to track traffic congestion via bottleneck analysis, thereby facilitating the early detection of significant congestion events. The discourse culminates in the exposition of a simulation model that integrates the bottleneck identification framework within urban traffic light control systems, demonstrating its superiority over existing conventional methodologies.

### (a) Jam Trees

The basic methodology presented here is based on Serok *et al*. [31] and introduces a real-time approach to monitor and rank urban traffic congestions based on their severity, measured in vehicle hours (VHs). Highlighting that non-recurrent congestions predominate, it underscores the need for efficient, real-time congestion detection. Our approach innovates by identifying emergent traffic bottlenecks without relying on historical patterns, thereby capturing both recurrent and non-recurrent congestions. It enables real-time tracking of all bottleneck dynamics, even during interventions like adaptive traffic light controls. Additionally, by assessing and prioritizing bottlenecks based on their impact on traffic flow, our method offers valuable insights for transportation system planning and congestion mitigation.

Serok *et al*. [31] introduced a novel method for the identification, cost evaluation and prioritization of urban traffic congestions and their origin in near-real time. This analysis was based on the spatiotemporal properties of congestion propagation. We started by defining a bottleneck as a link whose upstream is slower than its downstream. For our purposes, we defined upstream and downstream as applied both in space and time. In other words, we defined a street as a bottleneck if it were congested *before* its upstream, implying a probable *causality*. The innovative bottleneck identification methodology developed according to this concept simultaneously follows the evolution of every traffic congestion in the entire urban network and ranks all the traffic congestions based on their cost.

**Figure 1.**
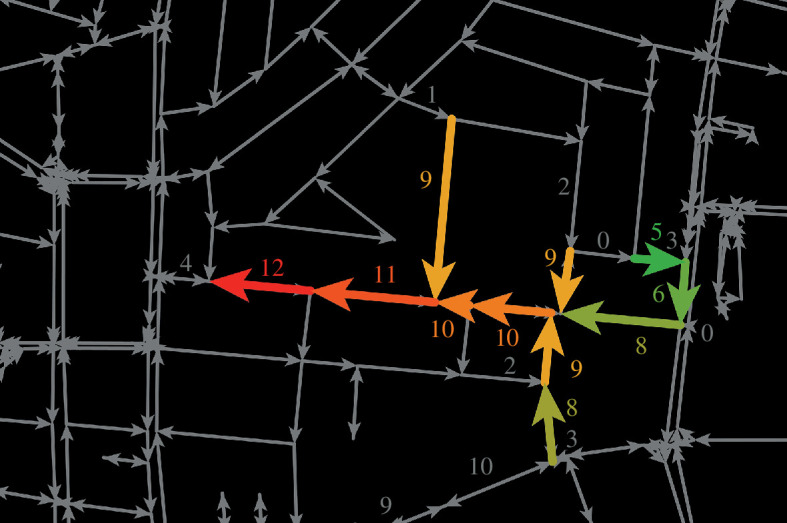
Clusters of traffic jams (TJs) are presented based on congestion duration in time units. A single TJ, with the red segment marking its trunk, has the longest congestion duration of 3 h, which indicates it was the initial congested street [31].

To identify traffic bottlenecks, we converted datasets of urban areas to dynamic, directed traffic networks where each node represented a junction, and each link represented a street segment between two junctions. The direction of the links represented the allowed direction of traffic on that street segment, and the weight of the link at time segment *t*, *W*(*t*), represents the temporal traffic relative speed, i.e. the ratio between the temporal speed and the speed at its maximal flow. We defined a street segment as congested at a specific time if *W*(*t*) < 1. To calculate $W\left(t\right)$ , we calculated the speed at the maximal flow of each link following a generalized car-following model that bridges microscopic and macroscopic models:


$$U^{1-m}=U_{f}^{1-m}\left[1-{\left(\frac{k}{k_{j}}\right)}^{l-1}\right],$$

where U represents the speed, $U_{f}$ represents the free-flow speed, k represents the density and $k_{j}$ represents traffic congestion density. We extracted $U_{f}$ as the 95% percentile of the maximal measured speed for every street segment. Then, we calculated k and q  (q is the Flow=k∗U) for each street and measurement. Next, we constructed for a specific time point t a new dynamic weighted network, *W*′(*t*), which is the cumulative continuous time, where each link has been considered as congested at *t*. Then we defined a process to create tree-shaped clusters of congested links, which we named JT. This was done by identifying the links with the highest weight *W*′, i.e. the links that have been congested the longest time, and defining them as potential trunks of a JT. The branches of the JT were identified by adding links or other trunks, connected to each trunk, with *W*′ ≤ *W*′_trunk_ with W ′≤θ where θ is a predefined maximal duration that a congested street segment is considered as the cause for the congestion in its upstream. By doing so, links that become congested shortly after the trunk are identified. This resulted in clusters that represent JTs and the time each of their links was loaded (figure 1).

While some traffic congestion may last many hours, their economic cost might be marginal, if, for example, they occurred in peripheral small streets. To assign prioritization for traffic congestion, we measured their cost in VHs. In order to calculate the cost of the JTs at different times and the links they include, we defined the cost of a link *Cij*(*t*) relative to its cost at free-flow speed. The link cost at time *t* represents the time it takes to cross a road (link) at time *t*, in comparison to the time it takes to cross this road in a maximal flow, multiplied by the number of drivers who crossed the endpoint of this link at a specific time. The momentary cost of a JT represents the sum of the costs of all the links that are included in the JT at a specific measured time, and the cumulative cost of a JT is the cost of the JT from the moment it was created until the time *t*.

The results of this framework and the analysis were demonstrated in Serok *et al*. [31] for London and Tel Aviv centres. The analysis of traffic congestion, based on the above methodology, distinguishes between macro-scale scaling characteristics and local meso-scale dynamics. While, at a macro level (i.e. PDFs of the cost and duration of the repetitive jam trees (RJTs)), the data align with scaling laws [73], a closer examination reveals variability in bottlenecks’ locations and timings, indicating diverse spatiotemporal behaviours at a meso-scale. Examining the meso-dynamics of traffic congestion reveals local shifts in bottleneck locations over time, highlighting the necessity for a big data-based urban transportation framework. The analysis of bottleneck repetition across different days showed a predominance of irregular occurrences, which means most bottlenecks did not recur daily.

Duration analysis parallels these findings, with longer-lasting bottlenecks recurring somewhat more often, yet only 10–15% of all bottlenecks repeat on 4–5 days, and the longest ones constitute <10%. Analysing the impact of measurement unit resolutions *T* on JT costs revealed a decrease in both total costs and their variance with larger *T* values, suggesting data smoothing and information loss over broader time intervals. This emphasizes the value of monitoring traffic dynamics at finer temporal resolutions.

Finally, when evaluating the number of distinct bottlenecks connected to each street involved in traffic congestion during the study period, it was found that streets were connected to a varying number of bottlenecks that ranged between 1 and 22, independent of their cost. This indicates that although traffic congestion may be localized to specific city areas or streets, the precise locations of causative bottlenecks fluctuate over time. This result suggests that constant, predefined solutions for traffic reduction cannot be the only solution to manage urban traffic congestion and strengthen the necessity of near real-time analysis based on big data to improve urban traffic flow. Based on our proposed method, when a trunk is dissolved, the tree can continue to exist with another branch acting as its trunk.

### (b) Spatiotemporal evolution of the traffic bottlenecks

In this section, we present a new framework for forecasting congestion, based on a novel framework that follows the network propagation and dissipation of TJs originating from bottleneck emergence, growth and recovery [70]. This approach demonstrates that the early stages of jam growth are highly correlated with the maximal size of the jam, providing a robust method for early congestion forecasting.

The methodology identified specific traffic bottlenecks and their spatiotemporal dynamics as the key to identifying heavy urban congestions in their early stages and before they reach their full size. This targeted approach to understanding and forecasting TJs represents a novel direction in traffic research, emphasizing the prevention of congestion through early detection of critical bottlenecks.

The identification of heavy congestions in their early stages is based on the initial growth speed of a JT, which was found to be highly correlated with the maximal size of the jam. Specifically, the method correlates the maximal size of congestion with the earliest attributes of congestion propagation, notably the initial growth speed, $V_{T_{i}}$ . This is represented by the equation:


$$V_{T_{i}\;}=\frac{S_{T_{i}}}{T_{i}}$$

where $S_{T_{i}}$ is the number of congested roads connected to the bottleneck at the chosen early growth duration $T_{i}$ (which is the time interval between the emergence of a bottleneck and a selected early time before the congestion component reaches its maximal size). The study found that the initial growth speed, even within the first 15 min of a jam, is highly correlated with the maximum size of the jam. This insight is pivotal for early detection and forecasting of heavy traffic congestions, providing a significant tool for urban traffic management to implement proactive measures before congestion escalates into large network-wide jams.

The article [70] empirically validates this methodology using traffic data from Beijing and Shenzhen, demonstrating that early growth speed is a reliable predictor for forecasting major bottlenecks. This methodological approach for identifying heavy congestions in their early stages represents a significant advancement in traffic management, offering the potential to improve urban traffic flow and reduce the impact of congestions through early detection and intervention, potentially reducing the incidence of heavy congestion and improving overall traffic conditions in urban areas.

While the study focuses on data from Beijing and Shenzhen, the framework it proposes has the potential for application in other urban settings globally. This universal framework enhances the article’s significance, suggesting that its findings could inform traffic management and congestion prevention strategies in cities worldwide.

### (c) Elaborating on the JT methodology

We further refined the methods to identify and prioritize bottlenecks, and also expanded the JT concept from [31], when analysing very extensive real-world data [71] to include more JT types and their characteristics. This advancement allows for scenarios where JTs intersect or share components, enhancing traffic analysis in complex urban networks. Our improved model offers better utility and accuracy for understanding urban traffic congestion, making traffic pattern analysis more effective for addressing megacity roadway challenges.

**Figure 2.**
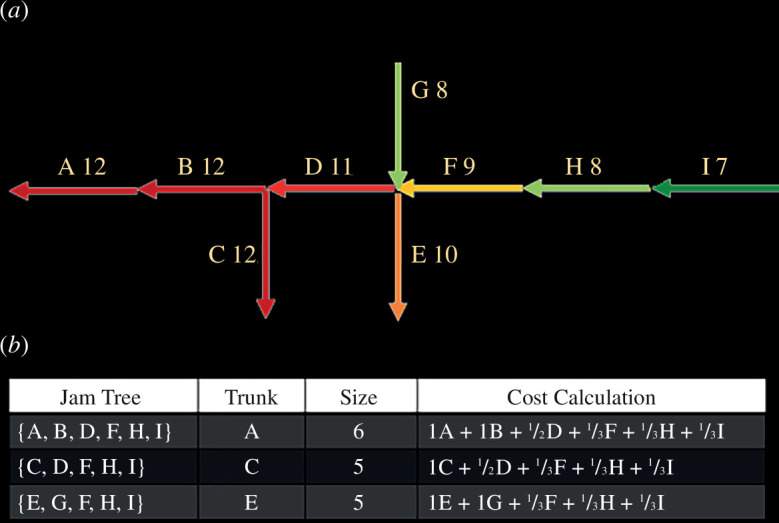
(*a*) Illustrates potential jam trees (JTs) where each arrowed link signifies a road segment and its traffic flow direction. The numbers above links show jam durations in time units. (*b*) Provides details on these JTs, including data on the main trunk, tree size and cost for each tree identified in (*a*). A JT is characterized by a singular trunk, its size by the total segment count (trunk plus branches) and its cost by the combined weighted duration of all its components [71].

The new approach for analysing traffic congestion patterns [71] is based on delineating road segments and their respective traffic flow directions. The jam duration for each link facilitates the computation of both the size and cost of a JT, where size denotes the number of road segments that a JT contains, and cost is derived as follows. If a segment links to multiple trunks, its cost is split equally, assuming each trunk has an equal chance of causing congestion (figure 2).

This expanded model introduces a more comprehensive and complete framework for quantifying traffic congestion’s spatial dynamics, accommodating complex scenarios like shared trunk–branch relationships, thereby refining the understanding of congestion propagation and its implications on urban traffic networks.

### (d) Developing a decentralized traffic control based on traffic bottlenecks

This section introduces a decentralized traffic light control method, the ‘Tree Method’, leveraging tree-shaped identification and cost analysis for phase prioritization amidst urban traffic’s feedback loops [72]. Recognizing the high costs of traffic-responsive signals, [72] utilized SUMO, an open-source traffic simulator, to evaluate control system performance through simulations. This approach showcases the Tree Method’s efficacy in real-time traffic optimization, demonstrating its advantages over conventional methods, particularly under heavy traffic conditions, using comparisons and simulation outcomes to underline its potential for enhancing urban traffic management.

The proposed ‘Tree Method,’ [72] grounded in self-organized dynamic congestion trees, operates on a ‘fixed cycle, fixed order’ principle, maintaining constant cycle lengths and phase orders, with phase durations determined at each cycle’s start. Its primary advantage lies in its selective activation capability, allowing for application at specific times or intersections to optimize traffic management. This approach avoids the pitfalls of unpredictable phase sequencing, which can cause driver frustration and unsafe driving conditions by maintaining predictable patterns. The method is underpinned by the concept of back pressure from temporary traffic bottlenecks, which occur when traffic supply and demand are misaligned during traffic light phases, potentially leading to widespread congestion. By identifying and evaluating the cost of congestion trees and their impact on traffic flow, including distant and higher hierarchy streets, the Tree Method adapts phase durations based on congestion cost assessments at cycle ends, employing a multi-stage methodology for refined traffic control.

The Tree Method was compared against the SUMO-actuated traffic light control method. This is a gap-based actuated method built into SUMO, which uses virtual detectors on each lane to determine the next phase and its duration. This method operates by extending traffic phases when a continuous flow of vehicles is detected. It transitions to the next phase once a sufficient time gap between vehicles is identified, enabling more balanced allocation of green time across phases and adjusting the cycle duration to accommodate changing traffic conditions.

Actuated methods are prominent in countries like Germany and England by 2010, and they dynamically adjust signals based on vehicle detection, offering superior flexibility over the uniform methods. This approach is particularly effective at reducing delays at intersections with variable traffic, adjusting green light durations within predefined limits. Its simplicity, minimal configuration needs and robust performance in fluctuating traffic conditions make it a benchmark in traffic management research [74,75].

These methods were evaluated using two different origin–destination (OD) matrices: one where the origins and destinations for each vehicle were chosen randomly and another based on real traffic volume data to generate probabilities for the origins and destinations, referred to as a more realistic approach. The results of the simulations show the effectiveness of the Tree Method in managing urban traffic through real-time signal control. The method’s performance was measured in terms of average travel time and throughput, utilizing the SUMO’s TraCI engine for simulations based on realistic and randomly generated OD matrices under different traffic loads. The Tree Method significantly outperformed all other methods tested, showing a 40% reduction in average travel time and a 35% improvement in throughput under high-traffic load scenarios with a random OD matrix. Under moderate load conditions, the method yielded over a 19% increase in throughput and a reduction in travel time by over 43%.

The simulations also highlighted the SUMO-actuated method’s limitations under high-load conditions, suggesting that the decentralized, dynamic approach, employed by the Tree Method, is more effective in managing heavy and conflicting traffic flows. The Tree Method’s superiority was further demonstrated in scenarios based on realistic OD matrices, where it achieved better results than the SUMO-actuated method by over 23% for high-load levels and by over 7% for moderate ones and reduced average travel time by 43 and 21%, respectively.

In a detailed comparison between the Tree Method and the SUMO-actuated method, the Tree Method consistently performed better across all driving times, particularly for short driving times where it significantly improved the fraction of drivers reaching their destinations within the simulation time. This analysis underlines the Tree Method’s capability to enhance traffic flow efficiency and reliability, proving its potential as a superior traffic management solution.

## 4. Discussion

The introduction of technological developments has significantly changed the role of urban planners. Traditionally, urban planning focused on static transportation planning and the physical layout of cities to influence the characteristics of different areas, using tools and methodologies that relied on direct, tangible impacts on urban environments. This approach allowed urban planners to craft the urban experience through the deliberate design of streets and public spaces, aiming to balance the needs of the community and the functional requirements of urban infrastructure.

The rise of digital technology, particularly in the realms of data collection, analysis and the internet, has introduced a new dimension to urban planning. The ability to track human movement through GPS and collect and analyse big real-time data provided by mobile applications like Waze and Google Maps has shifted the focus from static planning to dynamic, real-time management of urban environments. These technologies offer an updating information on traffic conditions, allowing for immediate adjustments to routes and transportation plans. This shift represents a move away from traditional planning’s top-down approach, where the planner’s vision directly shaped the urban landscape, to a more fluid, bottom-up approach influenced by the immediate needs and movements of city dwellers.

In fact, this technological shift has resulted in weakening the urban planner’s traditional role in directly influencing urban form and function through prescriptive design and planning. The use of navigation systems, for instance, has changed traffic flow patterns, with quiet residential streets now often serving as throughways to avoid congestion on main roads, undermining the original intent of urban design and planning.

Understanding the dynamic nature of urban traffic congestion requires innovative methodologies that transcend traditional analysis. In this vein, the JTs methodology, developed by Serok *et al*. [31], introduces a groundbreaking real-time approach to monitoring and evaluating urban traffic flow. By focusing on VH to quantify congestion severity, this approach differentiates between recurrent and non-recurrent congestions, offering a nuanced view of traffic dynamics. It pioneers in identifying emergent traffic bottlenecks without the dependency on historical patterns, thus capturing a comprehensive spectrum of congestion types.

This methodology shows that while overall traffic congestion patterns follow universal distributions, local factors significantly affect dynamics, indicating traffic bottlenecks rarely repeat daily. This reveals a complex layer beneath apparent stability, where bottlenecks’ spatial and temporal positions vary significantly. These findings suggest that there is a need to implement specialized solutions for the management of traffic congestions across diverse locations and temporal frames, with an emphasis on prioritizing interventions based on a real-time assessment of each bottleneck’s relative impact on the broader road network. As exemplars of complex networks, urban traffic systems consist of an array of components and their interactions, encompassing street connectivity, land use patterns, public transportation infrastructure and traffic light control mechanisms, among others. The multifaceted nature of these elements significantly complicates the task of forecasting urban traffic patterns. Consequently, modifications within the traffic light control system can precipitate unforeseen consequences throughout the urban matrix, exemplified by the potential for congestion alleviation in one locale to inadvertently catalyse congestion in alternative locations. This outlines the need to develop and deploy novel, dynamic and real-time solutions. Such solutions must leverage extensive big data analytics, encompassing the collection, analysis and application of data in real time (or near-real time) and their integration into operational urban systems. Such systems will continuously self-adjust in response to the system’s dynamics, offering progressively optimized solutions for mitigating urban traffic congestion.

A dynamic optimization process of this nature remains intricate and undeveloped, primarily due to the reciprocal causality existing between traffic lights and the actual flow of traffic. Nonetheless, in the context of the big data revolution, it is plausible to anticipate that, despite computational hurdles, the development of such solutions is feasible. However, the bottleneck identification methodology is a step towards this goal, as it simultaneously identifies all traffic bottlenecks as soon as they emerge; it allows to accurately follow their propagation in near-real time even while intervening in the system, for example, by using an adaptive traffic light control system. Therefore, this approach can potentially enhance current systems that utilize big data to identify traffic congestion.

The bottleneck approach was further enriched by the insights from Duan *et al*. [70], which offers a novel framework for forecasting congestion based on the early stages of jam growth (specifically within the initial 15 min window following the emergence of congestion). It implies that the predictive framework developed by Duan *et al*. [70] can provide an alerting indicator that could help urban planners improve urban traffic flow by addressing the sources of destructive TJs before they develop into their maximal size. In other words, a real-time traffic control system has the potential to address the source of the problem before it significantly impacts the upstream streets. This can be achieved by dynamically diverting traffic to alternative routes in accordance with urban planners’ guidance and considerations. This finding underscores the importance of real-time traffic management tools and their potential to empower urban planners.

Finally, we presented a first attempt to develop and evaluate an adaptive method of self-organized traffic control system [72]. This was done using a simulation model that is based on an extended definition of a JT, which includes more general cases where several JTs may overlap and share the same trunk or branches [71]. The JT was used to calculate the cost of congested trees for each intersection at the end of each cycle, and this cost was used to define the duration of each phase for the next cycle. The simulation utilized average travel time (in seconds) and throughput (number of vehicles throughout the simulation) as primary performance indicators, calculated via SUMO’s TraCI engine for vehicles reaching their destinations within the simulation. The Tree Method was found to notably surpass alternative approaches, specifically in realistic high-load scenarios. Further examination revealed the Tree Method’s superiority in both average and individual performances, benefiting the majority of drivers over time. It outperformed other methods by prioritizing traffic flows based on global rather than local costs, effectively addressing the root causes of congestion and its upstream effects.

This method’s advantage also lies in its simple analysis, allowing for real-time adjustments and complementing traffic flow feedback dynamics, making it highly applicable to real-world traffic systems. The Tree Method, which recalculates traffic trees at the end of each cycle without altering cycle duration, enables activation, particularly under high-load conditions, enhancing computational efficiency over time. These results suggest that an effective urban traffic light management system could negate the need for route optimization in navigation apps by eliminating the search for faster routes, with the potential for municipal operations to further incorporate urban road planning priorities.

Real-time urban traffic management responds to current conditions, highlighting smart city governance. This approach uses data not just for future improvements but for immediate actions, aiming to enhance both environmental sustainability and social fairness in cities. By allowing the residents to directly impact their environment and by giving additional power to city authorities, who often face delays in reacting to residents’ needs, the city becomes more adaptive, quickly adjusting to how people use it. This implies a shift in focus from long-term planning to taking immediate action. Such a shift must allow for a faster response to changes involving various city aspects that urban planners carefully consider and that are influenced by the city’s residents.

We claim that to regain their power and relevance, urban planners must adapt to this new technological landscape, which means leveraging the same real-time data and digital tools that have transformed urban mobility to inform more dynamic, responsive and integrated planning processes. The JT methodology enables planners to use real-time traffic and mobility data to understand how urban spaces are actually used rather than how they were intended to be used and, thus, adjust their plans accordingly. Moreover, urban planners can regain influence by positioning themselves as the intermediaries between the data-driven insights provided by technology and the long-term visions for sustainable, equitable and livable cities. This involves not only managing the immediate impacts of technology on urban mobility but also integrating these insights into broader urban development strategies that prioritize human well-being, environmental sustainability and social equity.

In essence, the role of urban planners is evolving from being the architects of physical spaces to the curators of urban ecosystems, where technology, data and human experience intersect. By embracing this role, urban planners can harness the power of technology to create more adaptable, resilient and human-centred urban environments.

## 5. Summary

This study leverages the conceptual framework of directed weighted networks to dissect urban traffic flow, aligning with recent research in strategic urban traffic management. Through a meticulous process of transforming urban datasets into dynamic, directed traffic networks and employing an innovative expansion of JTs, this methodology not only enhances the understanding of bottleneck dynamics but also quantifies the economic impact of congestion. The integration of temporal and spatial analyses, alongside cost assessments, propels forward the strategic planning and mitigation of urban traffic congestion. By offering a detailed comparison with existing traffic light control methods and elucidating the potential of decentralized traffic management, this study substantiates the efficacy of the JT methodology in improving urban traffic flow and underscores its universal applicability in diverse urban settings, showcasing a significant advancement in traffic management research.

The significance of this article extends beyond its immediate contributions to traffic management; it posits a nuanced understanding of traffic dynamics, coupled with advanced traffic management technologies, that can restore the influence of urban planning in the digital era. It fosters more liveable, equitable and efficient urban environments by suggesting that urban planning should be flexible and responsive, fostering a feedback loop that adapts to and influences the evolving urban environment. It highlights the potential to fundamentally alter how cities approach traffic management and urban planning, making urban environments more navigable, efficient and equitable.

## Data Availability

We published most of the research data and developed code for public use. They are available at: https://github.com/nimrodSerokTAU/bottlenecks-prioritization, https://zenodo.org/records/13824589, https://zenodo.org/records/13811786 and https://zenodo.org/records/13810838 [76–78].

## References

[1] Batty M , Cheshire J . 2011 Cities as flows, cities of flows. In Environment and planning B: planning and design, pp. 195–196, vol. **38** . London, England: SAGE Publications Sage UK. (10.1068/b3802ed)

[2] Banister D . 2005 Unsustainable transport: city transport in the new century. London, UK: Routledge.

[3] Hillier B . 1996 Cities as movement economies. Urban Des. Int. **1** , 41–60. (10.1057/udi.1996.5)

[4] Chase WG . 1983 Spatial representations of taxi drivers. In The acquisition of symbolic skills, pp. 391–405. Boston, MA: Springer.

[5] Kuipers B , Tecuci DG , Stankiewicz BJ . 2003 The skeleton in the cognitive map: a computational and empirical exploration. Environ. Behav. **35** , 81–106. (10.1177/0013916502238866)

[6] Barthélemy M . 2011 Spatial networks. Phys. Rep. **499** , 1–101. (10.1016/j.physrep.2010.11.002)

[7] Helbing D . 2003 A section-based queueing-theoretical traffic model for congestion and travel time analysis in networks. J. Phys. A. Math. Gen. **36** , L593–L598. (10.1088/0305-4470/36/46/L03)

[8] Gössling S . 2016 Urban transport justice. J. Transp. Geogr. **54** , 1–9. (10.1016/j.jtrangeo.2016.05.002)

[9] Martens K . 2012 Justice in transport as justice in accessibility: applying Walzer’s ‘Spheres of Justice’ to the transport sector. Transportation **39** , 1035–1053. (10.1007/s11116-012-9388-7)

[10] Backfrieder C , Ostermayer G , Mecklenbrauker CF . 2016 Increased traffic flow through node-based bottleneck prediction and V2X communication. IEEE trans. Intell. Transp. Syst. **18** , 349–363. (10.1109/TITS.2016.2573292)

[11] Kashinath SA *et al* . 2021 Review of data fusion methods for real-time and multi-sensor traffic flow analysis. IEEE Acces. **9** , 51258–51276. (10.1109/ACCESS.2021.3069770)

[12] Shi Q , Abdel-Aty M . 2015 Big data applications in real-time traffic operation and safety monitoring and improvement on urban expressways. Transp. Res. Part C Emerg. Technol. **58** , 380–394. (10.1016/j.trc.2015.02.022)

[13] Penn A , Hillier B , Banister D , Xu J . 1998 Configurational modelling of urban movement networks. Environ. Plann. B **25** , 59–84. (10.1068/b250059)

[14] Buldyrev SV , Parshani R , Paul G , Stanley HE , Havlin S . 2010 Catastrophic cascade of failures in interdependent networks. Nature **464** , 1025–1028. (10.1038/nature08932) 20393559

[15] Batty M . 2007 Cities and complexity: understanding cities with cellular automata, agent-based models, and fractals. The MIT press.

[16] Portugali J . 1997 Self-organizing cities. Futures **29** , 353–380. (10.1016/S0016-3287(97)00022-0)

[17] Batty M . 2016 Complexity in city systems understanding, evolution, and design. In A planner’s encounter with complexity, pp. 99–122. Routledge.

[18] Cervero R . 1998 The transit metropolis: a global inquiry. Island press.

[19] Lighthill MJ , Whitham GB . 1955 On kinematic waves II. a theory of traffic flow on long crowded roads. Proc. R. Soc. Lond. A Math. Phys. Sci. **229** , 317–345. (10.1098/rspa.1955.0089)

[20] Richards PI . 1956 Shock Waves on the highway. Oper. Res. **4** , 42–51. (10.1287/opre.4.1.42)

[21] Arnott R , Small K . 1994 The economics of traffic congestion. Am. Sci. **82** , 446–455.

[22] Daganzo CF . 2002 A behavioral theory of multi-lane traffic flow. Part II: merges and the onset of congestion. Transp. Res. Part B Methodol. **36** , 159–169. (10.1016/S0191-2615(00)00043-6)

[23] Hidas P . 2002 Modelling lane changing and merging in microscopic traffic simulation. Transp. Res. Part C Emerg. Technol. **10** , 351–371. (10.1016/S0968-090X(02)00026-8)

[24] Daganzo CF . 1994 The cell transmission model: a dynamic representation of highway traffic consistent with the hydrodynamic theory. Transp. Res. Part B Methodol. **28** , 269–287. (10.1016/0191-2615(94)90002-7)

[25] Daganzo CF . 1995 The cell transmission model, part II: Network traffic. Transp. Res. Part B Methodol **29** , 79–93. (10.1016/0191-2615(94)00022-R)

[26] Daqing L , Yinan J , Rui K , Havlin S . 2014 Spatial correlation analysis of cascading failures: congestions and blackouts. Sci. Rep. **4** , 5381. (10.1038/srep05381) 24946927 PMC4064325

[27] Wu JJ , Sun HJ , Gao ZY . 2007 Cascading failures on weighted urban traffic equilibrium networks. Physica A **386** , 407–413. (10.1016/j.physa.2007.08.034)

[28] Brockmann D , Helbing D . 2013 The hidden geometry of complex, network-driven contagion phenomena. Science **342** , 1337–1342. (10.1126/science.1245200) 24337289

[29] Saberi M *et al* . 2020 A simple contagion process describes spreading of traffic jams in urban networks. Nat. Commun. **11** , 1616. (10.1038/s41467-020-15353-2) 32265446 PMC7138808

[30] Nguyen H , Liu W , Chen F . 2016 Discovering congestion propagation patterns in spatio-temporal traffic data. IEEE Trans. Big Data **3** , 169–180. (10.1109/TBDATA.2016.2587669)

[31] Serok N , Havlin S , Blumenfeld Lieberthal E . 2022 Identification, cost evaluation, and prioritization of urban traffic congestions and their origin. Sci. Rep. **12** , 13026. (10.1038/s41598-022-17404-8) 35906267 PMC9338062

[32] Anbaroglu B , Heydecker B , Cheng T . 2014 Spatio-temporal clustering for non-recurrent traffic congestion detection on urban road networks. Transp. Res. Part C Emerg. Technol. **48** , 47–65. (10.1016/j.trc.2014.08.002)

[33] Avila AM , Mezić I . 2020 Data-driven analysis and forecasting of highway traffic dynamics. Nat. Commun. **11** , 2090. (10.1038/s41467-020-15582-5) 32350245 PMC7190853

[34] Hanna R , Kreindler G , Olken BA . 2017 Citywide effects of high-occupancy vehicle restrictions: evidence from 'three-in-one' in Jakarta. Science **357** , 89–93. (10.1126/science.aan2747) 28684524

[35] Wu Y , Tan H , Qin L , Ran B , Jiang Z . 2018 A hybrid deep learning based traffic flow prediction method and its understanding. Transp. Res. Part C Emerg. Technol. **90** , 166–180. (10.1016/j.trc.2018.03.001)

[36] Arnott R , Palma A , Lindsey R . 1993 A structural model of peak-period congestion: a traffic bottleneck with elastic demand. Am. Econ. Rev. 161–179.

[37] Huang HJ , Lam WHK . 2002 Modeling and solving the dynamic user equilibrium route and departure time choice problem in network with queues. Transp. Res. Part B Methodol. **36** , 253–273. (10.1016/S0191-2615(00)00049-7)

[38] Olmos LE , Çolak S , Shafiei S , Saberi M , González MC . 2018 Macroscopic dynamics and the collapse of urban traffic. Proc. Natl Acad. Sci. USA **115** , 12654–12661. (10.1073/pnas.1800474115) 30530677 PMC6294880

[39] Ran B , Boyce D . 2012 Modeling dynamic transportation networks: an intelligent transportation system oriented approach. Springer Science & Business Media.

[40] Yildirimoglu M , Ramezani M , Geroliminis N . 2015 Equilibrium analysis and route guidance in large-scale networks with MFD dynamics. Transp. Res. Procedia **9** , 185–204. (10.1016/j.trpro.2015.07.011)

[41] Kerner BS , Klenov SL . 2003 Microscopic theory of spatial-temporal congested traffic patterns at highway bottlenecks. Phys. Rev. E **68** , 36130. (10.1103/PhysRevE.68.036130) 14524855

[42] Papageorgiou M , Ben-Akiva M , Bottom J , Bovy PHL , Hoogendoorn SP , Hounsell NB *et al* . 2007 ITS and traffic management. In Handbooks in operations research and management science, pp. 715–774, vol. **14** . (10.1016/S0927-0507(06)14011-6)

[43] Sessions GM . 1971 Traffic devices: historical aspects thereof

[44] Hamilton A , Waterson B , Cherrett T , Robinson A , Snell I . 2013 The evolution of urban traffic control: changing policy and technology. Transp. Plann. Technol. **36** , 24–43. (10.1080/03081060.2012.745318)

[45] Bell MC , Bretherton RD . 1986 Ageing of fixed-time traffic signal plans. In International conference on road traffic control.

[46] Le T , Kovács P , Walton N , Vu HL , Andrew LLH , Hoogendoorn SSP . 2015 Decentralized signal control for urban road networks. Transp. Res. Part C Emerg. Technol. **58** , 431–450. (10.1016/j.trc.2014.11.009)

[47] Nowé A , Vrancx P , De Hauwere YM . 2012 Game theory and multi-agent reinforcement learning. In Adaptation, learning, and optimization. (10.1007/978-3-642-27645-3_14)

[48] Wei H , Xu N , Zhang H , Zheng G , Zang X , Chen C *et al* . 2019 Colight: learning network-level cooperation for traffic signal control. In International conference on information and knowledge management, proceedings. (10.1145/3357384.3357902)

[49] Thunig T , Kühnel N , Nagel K . 2019 Adaptive traffic signal control for real-world scenarios in agent-based transport simulations. Transp. Res. Procedia **37** , 481–488. (10.1016/j.trpro.2018.12.215)

[50] Cools SB , Gershenson C , D’Hooghe B . Self-organizing traffic lights: a realistic simulation. In Advances in applied self-organizing systems, p. 2013; (10.1007/978-1-4471-5113-5_3)

[51] Albert R , Jeong H , Barabási AL . 2000 Error and attack tolerance of complex networks. Nature **406** , 378–382. (10.1038/35019019) 10935628

[52] Cohen R , Havlin S . 2010 Complex networks: structure, robustness and function. Cambridge university press.

[53] Kurant M , Thiran P . 2006 Layered complex networks. Phys. Rev. Lett. **96** , 138701. (10.1103/PhysRevLett.96.138701) 16712049

[54] Rinaldi SM , Peerenboom JP , Kelly TK . 2001 Identifying, understanding, and analyzing critical infrastructure interdependencies. IEEE Control Syst. **21** , 11–25. (10.1109/37.969131)

[55] Gao J , Buldyrev SV , Stanley HE , Havlin S . 2012 Networks formed from interdependent networks. Nat. Phys. **8** , 40–48. (10.1038/nphys2180) 23005189

[56] Barrat A , Barthélemy M , Pastor-Satorras R , Vespignani A . 2004 The architecture of complex weighted networks. Proc. Natl Acad. Sci. USA **101** , 3747–3752. (10.1073/pnas.0400087101) 15007165 PMC374315

[57] Colizza V , Barrat A , Barthélemy M , Vespignani A . 2006 The role of the airline transportation network in the prediction and predictability of global epidemics. Proc. Natl Acad. Sci. USA **103** , 2015–2020. (10.1073/pnas.0510525103) 16461461 PMC1413717

[58] Daqing L , Kosmidis K , Bunde A , Havlin S . 2011 Dimension of spatially embedded networks. Nat. Phys. **7** , 481–484. (10.1038/nphys1932)

[59] Latora V , Marchiori M . 2002 Is the Boston subway a small-world network? Phys. A. Stat. Mech. Appl. **314** , 109–113. (10.1016/S0378-4371(02)01089-0)

[60] Chowell G , Hyman JM , Eubank S , Castillo-Chavez C . 2003 Scaling laws for the movement of people between locations in a large city. Phys. Rev. E **68** , 66102. (10.1103/PhysRevE.68.066102) 14754264

[61] Zaltz Austwick M , O’Brien O , Strano E , Viana M . 2013 The structure of spatial networks and communities in bicycle sharing systems. PLoS One **8** , e74685. (10.1371/journal.pone.0074685) 24040320 PMC3765359

[62] De Montis A , Barthélemy M , Chessa A , Vespignani A . 2007 The structure of interurban traffic: a weighted network analysis. Environ. Plann. B Plann Des. **34** , 905–924. (10.1068/b32128)

[63] Girvan M , Newman MEJ . 2002 Community structure in social and biological networks. Proc. Natl Acad. Sci. USA **99** , 7821–7826. (10.1073/pnas.122653799) 12060727 PMC122977

[64] Guimerà R , Mossa S , Turtschi A , Amaral LAN . 2005 The worldwide air transportation network: anomalous centrality, community structure, and cities global roles. Proc. Natl Acad. Sci. USA **102** , 7794–7799. (10.1073/pnas.0407994102) 15911778 PMC1142352

[65] Serrano MA , Boguñá M , Vespignani A . 2009 Extracting the multiscale backbone of complex weighted networks. Proc. Natl Acad. Sci. USA **106** , 6483–6488. (10.1073/pnas.0808904106) 19357301 PMC2672499

[66] Blumenfeld-Lieberthal E . 2009 The topology of transportation networks: a comparison between different economies. Netw. Spat. Econ. **9** , 427–458. (10.1007/s11067-008-9067-6)

[67] Chen Y , Yan P , Zheng Z , Chen D . Identifying traffic bottleneck in urban road networks via causal inference. In Security, privacy, and anonymity in computation, communication, and storage: SpaCCS 2020 international workshops Nanjing, China, pp. 372–383. (10.1007/978-3-030-68884-4_31)

[68] Lee WH , Tseng SS , Shieh JL , Chen HH . 2011 Discovering traffic bottlenecks in an urban network by spatiotemporal data mining on location-based services. IEEE trans. Intell. Transp. Syst. **12** , 1047–1056. (10.1109/TITS.2011.2144586)

[69] Li D , Fu B , Wang Y , Lu G , Berezin Y , Stanley HE , Havlin S . 2015 Percolation transition in dynamical traffic network with evolving critical bottlenecks. Proc. Natl Acad. Sci. USA **112** , 669–672. (10.1073/pnas.1419185112) 25552558 PMC4311803

[70] Duan J , Zeng G , Serok N , Li D , Lieberthal EB , Huang HJ , Havlin S . 2023 Spatiotemporal dynamics of traffic bottlenecks yields an early signal of heavy congestions. Nat. Commun. **14** , 8002. (10.1038/s41467-023-43591-7) 38049413 PMC10695996

[71] Zeng G , Nimrod S , Blumenfeld Lieberthal E , Jinxiao D , Shiyan L , Shaobo S . 2024 Unveiling city jam-prints of urban traffic based on jam patterns. arXiv.

[72] Serok N , Havlin S , Lieberthal EB . 2023 Enhancing traffic flow efficiency through an innovative decentralized traffic control based on traffic bottlenecks. arXiv.

[73] Lopez C , Leclercq L , Krishnakumari P , Chiabaut N , van Lint H . 2017 Revealing the day-to-day regularity of urban congestion patterns with 3D speed maps. Sci. Rep. **7** , 14029. (10.1038/s41598-017-14237-8) 29070859 PMC5656590

[74] Aslani M , Mesgari MS , Wiering M . 2017 Adaptive traffic signal control with actor-critic methods in a real-world traffic network with different traffic disruption events. Transp. Res. Part C Emerg. Technol. **85** , 732–752. (10.1016/j.trc.2017.09.020)

[75] Tettamanti T , Mohammadi A , Asadi H , Varga I . 2017 A two-level urban traffic control for autonomous vehicles to improve network-wide performance. Transp. Res. Procedia **27** , 913–920. (10.1016/j.trpro.2017.12.160)

[76] Jinxiao D . 2023 DynamicsOfBottlencks.Github https://github.com/JinxiaoDuan/DynamicsOfBottlencks

[77] Nimrod S . 2023 Decentralized-traffic-bottlenecks.Github https://github.com/nimrodSerokTAU/decentralized-traffic-bottlenecks/

[78] Nimrod S . 2023 Decentralized-traffic-bottlenecks. Github https://github.com/nimrodSerokTAU/bottlenecks-prioritization

